# Epidemiology and Control of African Swine Fever in Vietnam: A Scoping Review

**DOI:** 10.3390/pathogens14040329

**Published:** 2025-03-29

**Authors:** Vo Dinh Chuong, Rachel A. Schambow, Nguyen Thi Diep, Phan Quang Minh, Nguyen Van Long, Bui Thi To Nga, Andres M. Perez

**Affiliations:** 1 Vietnam Department of Animal Health (DAH), Hanoi 10000, Vietnam; diep.dahvn@gmail.com (N.T.D.); phanquangminh1@gmail.com (P.Q.M.); long.dahvn@gmail.com (N.V.L.); 2 Center for Animal Health and Food Safety, College of Veterinary Medicine, University of Minnesota, Saint Paul, MN 55108, USA; scham083@umn.edu (R.A.S.); aperez@umn.edu (A.M.P.); 3 College of Veterinary Medicine, Vietnam National University of Agriculture (VNUA), Hanoi 10000, Vietnam; buitonga@gmail.com

**Keywords:** African swine fever, Vietnam, swine industry, biosecurity, live-attenuated vaccine, disease management, epidemiology

## Abstract

African swine fever (ASF) has had a devastating impact on Vietnam’s swine industry since its introduction in Vietnam in 2019, leading to the culling of six million pigs. This paper aimed to review the epidemiological dynamics of ASF in Vietnam and measures applied to control the disease. ASF progressed through an initial epidemic phase (2019–2020) and has transitioned into a more endemic phase (2021–2024). The disease spread rapidly during the epidemic phase, driven by human-mediated transmission routes and inadequate biosecurity practices, particularly on smallholder farms. To control ASF, the Vietnamese government endorsed a national control plan that included biosecurity enhancements, disease surveillance, establishing ASF-free compartments, researching and evaluating ASF vaccines, and strengthening the capacity of veterinary services. While these measures have helped reduce the number of outbreaks, challenges persist, including the emergence of recombinant ASF strains, limited vaccine adoption, and gaps in the veterinary infrastructure. ASF has substantially changed Vietnam’s swine industry, shifting toward reducing small-scale household farming and increasing professional households and large-scale farms. As ASF has transitioned into an endemic phase, sustainable strategies focusing on continuous monitoring, improved vaccination coverage, and education programs are essential in order to mitigate its impacts and ensure the resilience of Vietnam’s swine industry.

## 1. Introduction

The African swine fever virus (ASFV) is part of the *Asfivirus* genus within the *Asfarviridae* family. It carries a double-stranded DNA genome ranging from 170 to 193 kilobases in length. ASFV has been characterized into 24 distinct genotypes, most of which have been found in Africa [1], with only genotypes I and II having caused significant outbreaks outside the continent [2,3,4]. Swine species within the *Suidae* family, including both domestic and wild pigs, are susceptible to ASFV. The virus is transmitted through direct contact with infected pigs and indirect contact, such as via the consumption of contaminated feed or exposure to contaminated fomites [5,6]. Both the long- and short-distance transmission of ASFV has been mediated by human activities, particularly the movement of infected pigs and feeding infected pork products in swill or garbage feeding, as the virus can survive for long periods in pork products [7]. Additionally, *Ornithodoros* ticks serve as natural reservoirs for ASFV and can transmit the virus to pigs through blood-feeding [7,8]. ASFV causes a hemorrhagic syndrome in the affected swine with mortality rates that can reach up to 100%, depending on factors such as the host, virus dose, and transmission routes [9]. ASFV does not infect humans or other animal species [10]. The disease is reportable to the World Organisation for Animal Health (WOAH, founded as the OIE) and can have significant economic impacts on the affected countries, disrupting the international pig and pork trade and reducing pig production in affected areas. Consequently, ASF seriously threatens the global swine industry [11].

In recent years, ASF has expanded globally outside of Africa. In 2007, it spread to Eastern Europe, starting in the Caucasus region of Georgia. From there, the virus gradually expanded to the neighboring countries, including Armenia, Azerbaijan, Russia, and Belarus, impacting both domestic pigs and wild boars. In 2014, the first outbreak of ASF was reported within the European Union (EU), and, since then, numerous EU countries have experienced outbreaks [12]. In 2018, ASF was detected in China [13,14], and, since then, it quickly spread to many countries in Asia and the Pacific, including Mongolia, Vietnam, Cambodia, Democratic People’s Republic of Korea, Lao People’s Democratic Republic, Myanmar, The Philippines, Republic of Korea, Timor-Leste, Indonesia, Papua New Guinea, India, Malaysia, Bhutan, Thailand, Nepal, Singapore, Bangladesh, and Sri Lanka [15]. Additionally, in July 2021, ASF re-emerged in the Americas after nearly 40 years, first being detected in the Dominican Republic and subsequently in neighboring Haiti [12].

On 1 February 2019, the first outbreak of ASF in Vietnam was detected on a backyard pig farm in the Trung Nghia commune, Hung Yen City, in the northern province of Hung Yen. In Vietnam, an outbreak is defined as a commune where at least one pig is confirmed as infected with the ASF virus by laboratory testing (RT-PCR) [16]; hence, in this paper, the term “outbreaks” means commune-level outbreaks. As the Vietnam Department of Animal Health (DAH) reported, by December 2019 (less than one year since the first report), ASF had impacted 8553 out of 10,614 communes across 667 of 705 districts in all 63 provinces. Nearly 6 million pigs died or were culled, representing approximately 9% of the total pig population in the country. However, after 2019, the shift in ASF’s epidemiological status to endemic has become increasingly evident since its initial outbreak [17]. In addition, recently, ASFV strains with recombinant regions from both genotype I and II (rASFV I/II) were detected in Vietnam, raising concerns about their potential impact on mitigation measures in the country [18]. This shift and new recombinant strains of ASFV raise serious concerns about the challenges of managing endemic ASF and the future of Vietnam’s pig industry. This paper aims to review the epidemiological situation of ASF in Vietnam over the past six years, focusing on its transition from an epidemic to an endemic phase and the mitigation measures implemented during this period.

## 2. Methods

This scoping review was conducted according to the Preferred Reporting Items for Systematic Reviews and Meta-Analyses Extension for Scoping Reviews, PRISMA-ScR, guidelines [19]. The protocol for this scoping review has been registered in the OSF Registries database and is publicly accessible at https://doi.org/10.17605/OSF.IO/RBJ8V (accessed on 11 February 2025).

### 2.1. Eligibility Criteria

Papers were included in the screening process if they were conducted on ASF outbreaks in Vietnam, focusing on epidemiological studies (risk factors, transmission dynamics, and epidemiological factors affecting disease spread), mitigation strategies implemented in Vietnam to deal with ASF outbreaks, including ASF vaccine research, development, and experimental use, and written in English and Vietnamese. Studies were excluded if they were not performed in Vietnam and were not related to the focus of this review, as mentioned above.

### 2.2. Information Sources and Search Strategies

We searched three academic databases, PubMed, CAB Abstracts, and Agricola, using broad key search terms: “African swine fever” OR “ASF” AND “Vietnam”. We also used reports and grey literature available from the Ministry of Agriculture and Rural Development (MARD), DAH, Department of Livestock and Production (DLP), General Statistics Office (GSO), AgroMonitor, United States Department of Agriculture (USDA), Food and Agriculture Organisation of the United Nations (FAO), and WOAH.

### 2.3. Record Screening and Data Extraction

Records identified from databases were exported into Microsoft Excel 365 to facilitate duplication removal, initial screening, and ongoing tracking throughout the review process. Duplicates were identified and removed using Excel’s ‘Remove Duplicates’ function. Titles and abstracts of the remaining publications were also imported into Zotero, version 6.0.36, for further review. These records were screened for relevance by reviewing titles and abstracts against the eligibility criteria.

ASF outbreaks from 2019 to 2024 were used to generate distributed maps using the ArcGIS software (Pro 4.6.3.23467) (ESRI Inc., Redlands, CA, USA).

### 2.4. Synthesis of Results

Data were synthesized according to areas of interest, including the impacts of the ASF outbreak, ASF epidemiology (risk factors, transmission dynamics, and epidemiological factors affecting disease spread), mitigation strategies implemented in Vietnam to deal with ASF outbreaks, and ASF vaccine research, development, and experimental use.

## 3. Results

### 3.1. Screening Results

**Figure 1:**
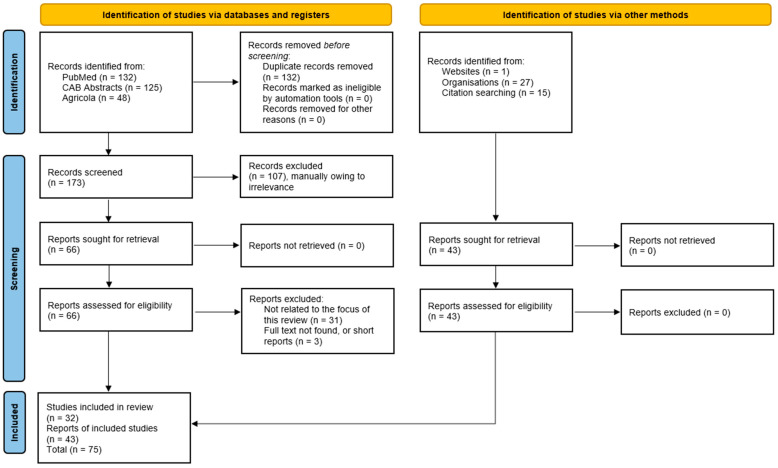
Schematic PRISMA-ScR flow chart for included studies.

In total, 305 records from PubMed (132), Agricola (48), and CAB Abstracts (125) were identified. First, 132 duplicates between databases were removed. Then, titles and abstracts of records were reviewed, of which 107 papers were removed due to being irrelevant or because the full text was unavailable. An additional 34 papers were removed for being not relevant after the full text was reviewed. In total, after screening, 32 papers in the relevant fields were reviewed. Grey literature includes conference presentations/reports (7) and regulatory documents (laws: 2, resolutions: 2, decrees: 3, decisions: 2, and official dispatches: 3) (Figure 1).

### 3.2. Overview of the Epidemiological Situation of ASF in Vietnam

#### 3.2.1. Initial Introduction and Epidemic Situation (February 2019 to December 2020)

Following the detection of ASF in China in early August 2018, the MARD issued an Emergency Action Plan to respond to ASF (Decision No. 4527/QĐ-BNN-TY dated 15 November 2018). Its objectives included preventing the disease from entering Vietnam, strict monitoring, early detection, and effective containment measures to minimize the economic, social, and environmental impacts if ASF were detected. The plan outlined two scenarios: (1) pre-emptive measures before ASF entered Vietnam and (2) response protocols upon detection. The plan emphasized interagency cooperation, resource allocation, public awareness, and compliance with national and international regulations to effectively manage and mitigate ASF risks. Simultaneously, MARD organized a simulation for these scenarios in Lao Cai province, which shares a border with China.

**Figure 2:**
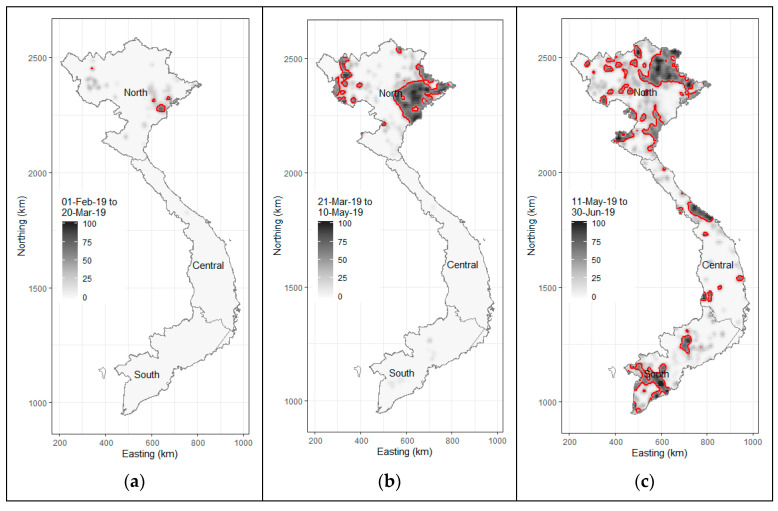
African swine fever in Vietnam, February to June 2019. Image plots showing the number of ASF-infected communes per square kilometer: (**a**) 1 February to 20 March; (**b**) 21 March to 10 May; and (**c**) 11 May to 30 June. Contour lines on each plot show areas where there were more than 40 ASF-positive communes per 100 communes per square kilometer.

Despite the proactive response from the Vietnamese government to prevent the incursion of the disease, on 19 February 2019, Vietnam confirmed its first ASF case at a backyard pig farm in Hung Yen province, located in the northern region of the country. The clinical signs and lesions observed in the affected pigs were consistent with those reported in prior ASF outbreaks in China and Georgia [20,21]. Simultaneously, ASF outbreaks were reported in five additional northern provinces. By March 2019, 17 provinces in the northern and north-central regions had recorded numerous ASFV-infected farms. By April 2019, Dong Nai province, known as the pig farming capital of southern Vietnam, along with four other provinces, also reported ASF cases. The ASF epidemic in Vietnam peaked swiftly in May 2019, with over 24 provinces reporting cases (Figure 2). Ninh Thuan, the province with the country’s smallest pig herd, was the last to report ASF outbreaks in August 2019. ASFV led to high infection and mortality rates among domestic pigs, with the virus spreading rapidly across multiple outbreaks in both open-access (where pigs are exposed to outdoor conditions) and confinement (where pigs are isolated from external environments) farm systems [10].

**Figure 3:**
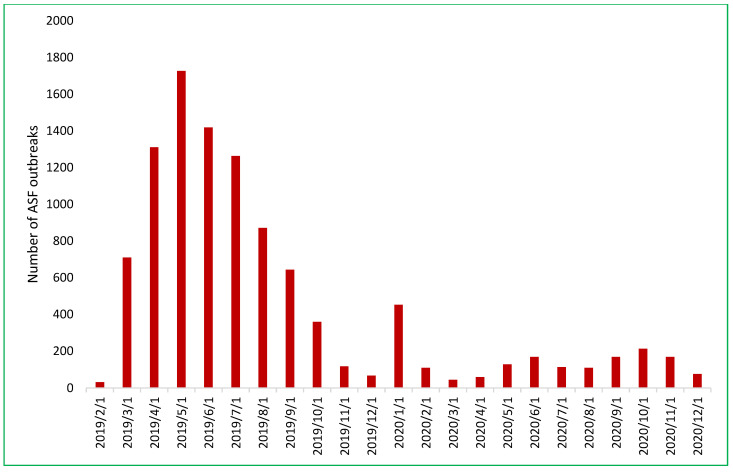
The number of African swine fever outbreaks in Vietnam by month from February 2019 to December 2020.

By December 2019, ASF had impacted 8553 out of 10,614 communes across 667 of 705 districts in all 63 provinces (Figure 3). Its initial spread in 2019 was characterized by a northeast-to-southeast trajectory beginning in Thai Binh province and concluding in Ninh Thuan province [22,23,24]. In 2020, there was a sharp reduction to 1569 outbreaks in 50/63 provinces. Throughout 2020, ASF exhibited more random outbreak patterns and less directional spread. High-risk spatiotemporal clusters were predominantly concentrated in the northern provinces [24].

During the initial phase of the ASF epidemic, nearly 6 million pigs died or were culled. This represented approximately a 25% decrease in the total pig population by December 2019 compared to 2018 [25], with an estimated economic cost exceeding 13,232 billion Vietnam Dong (equivalent to US$ 573 million) [16]. Poor biosecurity in smallholder farms (which account for 70% of pig production in Vietnam) was the main contributing factor to the rapid spread across the country over a short period, resulting in huge economic losses for the pig industry [26]. The acute form of the disease was observed during the first outbreak of ASF in Vietnam in 2019, with high mortality and case-fatality rates [27]. Genetic studies have shown that ASFV strains circulating in Vietnam from 2019 to 2020 were genotype II, displaying a complete genetic similarity with Chinese ASFV strains. This similarity suggests that the virus may have entered Vietnam from China through the uncontrolled cross-border trade of live pigs and pork [21,22,28,29,30,31].

#### 3.2.2. The Second Phase of the Outbreak (January 2021 to December 2023)

**Figure 4:**
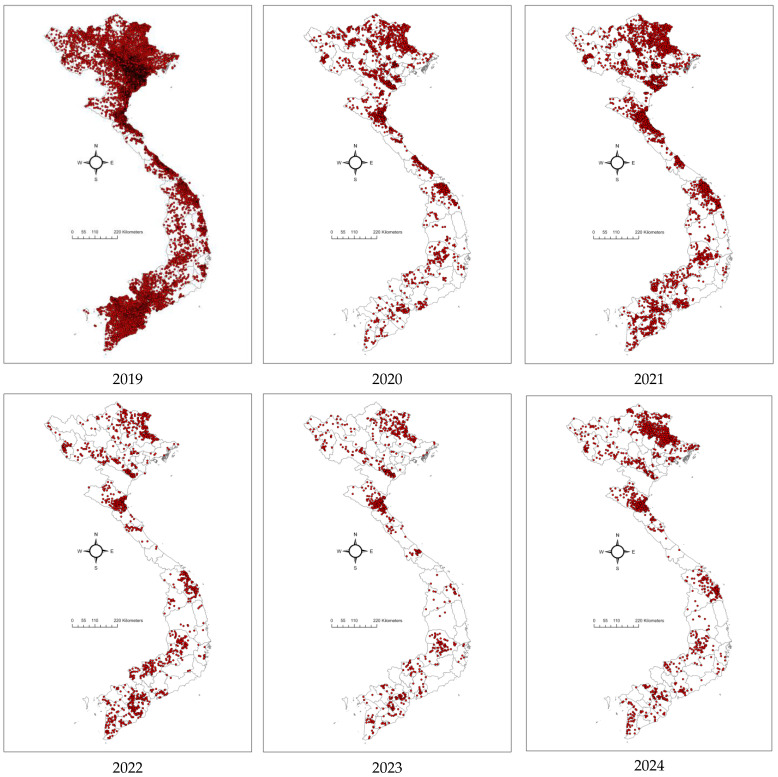
Maps showing the distributions of ASF outbreaks in Vietnam from 2019 to 2024. Red dots presented ASF outbreaks at the commune level.

From 2021–2023, the ASF outbreaks in Vietnam lacked any clear spatial pattern, though they remained concentrated in the northern provinces [24]. The number of outbreaks increased substantially in 2021, almost double compared to 2020 (1569 outbreaks), with 3029 outbreaks reported in 59/63 provinces. This later decreased to 1229 in 53/63 provinces in 2022. The figure reduced significantly in 2023, with 952 outbreaks in 46/63 provinces (Figure 4). Reports from provincial sub-Departments of Animal Health (SDAHs) and the Vietnam Animal Health Information System (VAHIS) [32] showed that most outbreaks occurred in small areas and were controlled by the localities. The spatiotemporal analysis identified significant clusters in January–April 2021 in four central provinces and in February 2022 in the southern provinces, marking a shift in the epicenter of outbreaks. These patterns reflect the evolving dynamics of ASF transmission after its introduction, transitioning from the more directed spread observed in earlier phases to a more widespread and less predictable pattern and a more endemic state [17].

#### 3.2.3. ASF Situation in Vietnam in 2024

From 1 January to 31 December 2024, 1669 ASF outbreaks have been reported in 48/63 provinces. Of these, 1652 were reported as resolved (passed 21 days from the onset), and 44 outbreaks in 17 provinces are reported as ongoing/active outbreaks (within 21 days from the last culling) [32]. Both resolved and ongoing outbreaks were distributed across the country. Northern Vietnam, particularly in the Red River Delta region, showed a high concentration of resolved outbreaks, reflecting effective but temporary containment measures, as recurring active cases remain a concern. In contrast, the Mekong Delta in southern Vietnam has been a significant hotspot, with numerous ongoing outbreaks indicating difficulties in controlling ASF in dense pig-farming regions. Central Vietnam has exhibited fewer overall outbreaks, but sporadic active outbreaks have still been detected.

Sequencing data from DAH showed that 60% of the ASF strains collected from northern provinces in 2024 carry both the B646L (p72) and E183 (p54) genes belonging to genotype II. The remaining 40% have the B646L (p72) gene belonging to genotype I and the E183L (p54) gene belonging to genotype II. These recombinant ASF strains in Vietnam are 100% identical to the recombinant strains that emerged in China in 2021–2022, Mongolia in 2022, and Cameroon in 2023 [33]. The cumulative ASF outbreaks in Vietnam from 2019 to 2024 are summarized in Table 1.

### 3.3. Transmission Dynamics and Epidemiological Factors Affecting Disease Spread

Studies have indicated that human-mediated factors primarily drive ASF transmission in Vietnam. These have included swill feeding, human/vehicle movements, and poor disinfection practices, accounting for 70–80% of farm-to-farm spread [22,26]. Significant associations were also identified between ASF outbreaks and various farm management practices and social factors, including the production type, the adherence to all-in-all-out policies, the use of insect nets, the transport trucks from slaughterhouses, and the water sourced directly from irrigation systems [34]. Risk factors also include the presence of workers, the absence of dressing rooms, wearing work clothes outside the farm, manure application practices, use of human food for pigs, and failure to quarantine visitors for 24 h before farm entry [34]. In addition, the distances from the affected farm to another farm within 500 m, proximity to irrigation systems within 200 m, poor hygienic practices by individuals within the farm, and inadequate hygiene at pig loading and unloading locations were identified as risk factors for ASF outbreaks [34]. Moreover, the farms located closer to main roads (<1000 m) and at lower elevations (<500 m) had dramatically higher risks for ASF outbreaks [35].

The level of biosecurity application is strongly influenced by farmers’ knowledge, attitudes, and prior experience in managing diseases [36]. Farmers with a greater awareness of and positive attitudes toward biosecurity measures and those with previous experience in disease outbreaks were more likely to implement effective biosecurity practices on their farms. Additionally, farms operated by individuals with over 10 years of pig-raising experience were 2.83 times more likely to experience an ASF outbreak compared to those with 5 to 10 years of experience, while farms, where farmers or agro-vet drug store employees managed veterinary care, faced significantly higher risks, with 5.33- and 5.70-times greater odds of ASF outbreaks, respectively, compared to farms under the care of professional veterinarians [34].

Animal density and management practices influenced ASF’s further spread in Vietnam. Barns located less than 10 m from the living quarters were associated with an 11.14-fold increase in the odds of an ASF outbreak compared to barns situated more than 50 m away. Among communes positive for ASF in Can Tho province (known as a swine production hub in the Mekong Delta), the within-commune farm-level incidence risk varied widely, ranging from 4.95–100%, with an incidence of 46 ASF-positive farms for every 100 farms at risk [37]. Delays in culling exacerbate infections, as shown by models where culling at 6–16 weeks reduced the median infected farms by up to 100% [26].

Several studies have also shown the regional and farm-scale variability of ASF transmission dynamics. A study across three northern provinces in Vietnam estimated the basic reproduction number (R_0_) using three methods: Exponential Growth (EG, which happens when a value increases over time following an exponential function, with its growth rate being directly proportional to its current magnitude [38,39]), Maximum Likelihood Estimation (ML, which is a statistical technique that utilizes a sample to infer the parameters of the underlying probability distribution responsible for generating the observed data [40]), and Attack Rate (AR, which is defined as the proportion of disease-free individuals developing a given disease over a specified time [41,42]). R_0_ values ranged between 1.01–2.32, with smaller farms (100–299 pigs) exhibiting higher R_0_ values and earlier infection peaks (days 30–60) compared to larger farms (300–999 pigs). ML-derived values, prioritized for worst-case scenarios, showed a rapid spread on smaller farms, with transmission rates (β) ranging between 0.16 and 0.37 [43]. Another study showed R_0_ increases with infectious duration, peaking at 10.8 in Thai Binh province for a 30-day infectious period, while national-level estimates remained lower. Thai Binh and Hung Yen provinces consistently reported the highest R_0_ values [44]. ASF transmission dynamics differed between sows and fattening pigs on commercial farrow-to-finish farms. R_0_ values for fattening pigs were significantly higher (3.8–4.76) compared to sows (1.55–1.78), thought to be driven by group housing and faster transmission rates [45]. An experiment of 15 pigs (10 experimental and 5 negative control) showed that the R_0_ values for EG and MLE were 2.916 and 4.015, respectively. In addition, the transmission rates (β) were estimated to be 0.729 for EG and 1.004 for MLE [46]. Another study evaluated the recovery of 14 gilts acutely infected with ASFV, revealing that all convalescent animals developed long-lasting high serum antibody levels without persistent viremia and did not excrete the virus through nasal discharge post-recovery [47]. Furthermore, there was no evidence of carrier status or disease recurrence in the recovered pigs or their offspring following the acute ASF outbreak. Table 2 summarizes studies related to the transmission of ASFV in Vietnam.

### 3.4. Impact of ASF on the Structure of Vietnam’s Swine Industry

**Figure 5:**
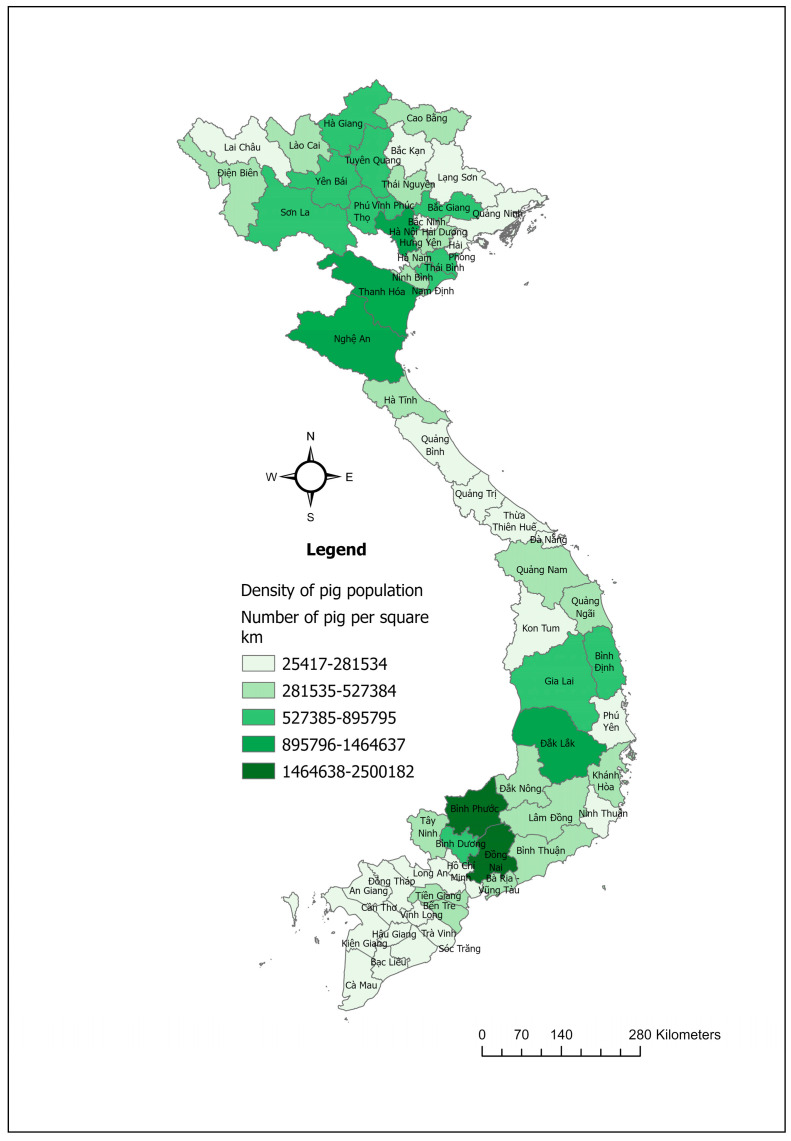
Maps showing the pig population density in Vietnam in 2023.

The swine industry in Vietnam is a major component of the country’s agricultural sector, with pork being the primary meat produced and consumed, making up around 64% of total meat consumption [48]. In 2023, Vietnam was recognized as a prominent country in pig farming, ranking fifth in the world in terms of total pig population (Figure 5) and sixth in pork production [49].

Livestock farming in Vietnam is categorized into farm-based and household livestock farming (or backyard farming). The majority of farms are family backyard operations, typically raising 4 to 10 pigs each and collectively contributing approximately 80% of the nation’s total pig production [50]. Farm-based livestock farming is classified into three main categories, including large-scale, medium-scale, and small-scale farms (Animal Husbandry Law, 2018). Decree No. 13/2020/NĐ-CP (21 January 2020) defined the scale of livestock farming as follows: household farming is under 10 livestock units (1 livestock unit is equivalent to 500 kg live animals); small-scale farm livestock farming ranges from 10 to under 30 livestock units; medium-scale farm livestock farming ranges from 30 to under 300 livestock units; and large-scale farm livestock farming is 300 livestock units or more. Most commercial pig farms in Vietnam are mid-sized operations, generally housing several hundred to several thousand pigs. These farms are frequently managed independently by farmers or under contracts with private companies. Medium-sized farms mainly serve as suppliers and have strong connections with smaller farms, although it is rare for pigs from small farms to be moved to farms of different sizes within the local vicinity [22,45].

**Figure 6:**
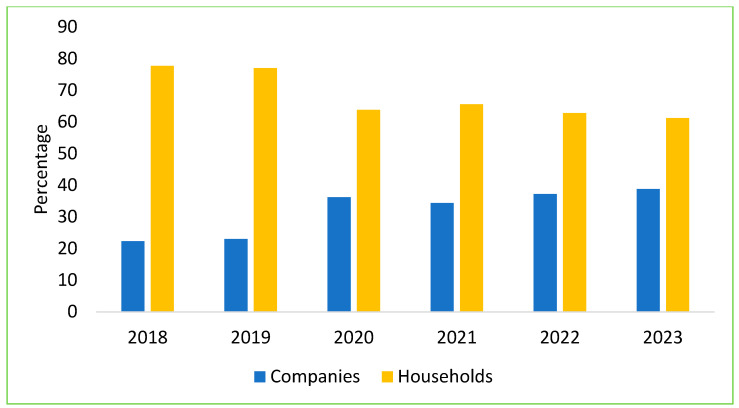
The structure of sow herd by scale in Vietnam from 2018 to 2023.

Over the past six years of the ASF outbreaks, livestock farming in Vietnam has significantly shifted toward reducing small-scale household farming and increasing professional households and large-scale farms, with a decrease in household farming by 5–7% per year. Specifically, from 2019 to 2022, small-scale household farms dropped by 15–20%. Currently, the output of pigs from small-scale household farms has decreased to 35–40%, while professional households and large-scale farms now account for 60–65% of pig production [51]. In 2023, the total live pig meat output nationwide reached over 4.8 million tons, an increase of 6.7% compared to 2022. Consequently, the industry is now driven by medium-to-large commercial farms operating independently or under contracts with private companies. In 2023, the total number of sows nationwide in Vietnam reached over 3.12 million (Figure 6), accounting for approximately 12% of the total pig herd, with an increase of 3.3% compared to the same period in 2022 [49]; approximately 38.8% of the country’s sow herd was contributed by company-owned farms, with both foreign direct investment (FDI) and domestic firms responsible for around 62% of total pork production [52].

**Figure 7:**
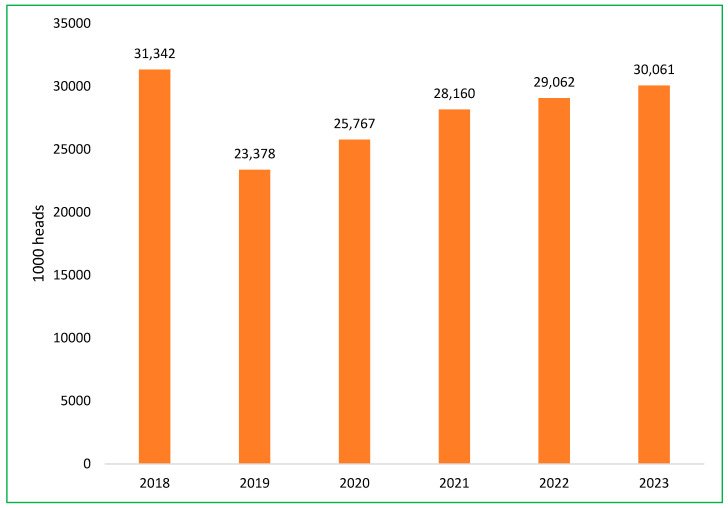
The change in the total swine population in Vietnam from 2018 to 2023.

The Vietnamese swine sector is currently experiencing a recovery, with an annual growth rate of 6.3% in pig herds, as efforts to rebuild following the impact of ASF continue [49]. The total pig population at the end of 2023 was more than 30 million head, approximately equal to 2018, before the ASF epidemic [52]. The impact of the ASF outbreak on the total swine population in Vietnam is depicted in Figure 7 [48,52]. The government’s role, combined with private sector investments, aims to strengthen biosecurity, improve production standards, and establish disease-resistant breeding practices to safeguard the industry against future outbreaks.

### 3.5. Strategies Implemented to Mitigate the Impacts of ASF

#### 3.5.1. Initial Response to ASF

Following the first detection of ASF in February 2019, the Vietnamese government established the National Steering Committee on ASF prevention and control in March 2019. The committee issued guidelines for implementing ASF prevention measures and guided the restocking of pig herds for stakeholders and producers. Key initiatives included Resolution No. 16/NQ-CP (dated 7 March 2019), Resolution No. 42/NQ-CP (dated 18 June 2019), and Decision No. 793/QĐ-TTg (dated 27 June 2019), which outlined urgent solutions for ASF prevention and control with specified mechanisms and policies to support funding for disease prevention, including compensation for producers/farmers who were required to depopulate pigs due to ASF (based on Decree No. 02/2017/NĐ-CP dated 9 January 2017) [53,54]. MARD also issued many official letters in a timely manner to regulate and adjust updated solutions to prevent the spread of the virus. Specifically, on 7 July 2019, the Prime Minister endorsed the “National Plan for Prevention and Control of African Swine Fever, Period 2020 to 2025” (Decision No. 972/QĐ-TTg), which highlighted 13 strategies for the prevention and control of ASF. These included enhanced biosecurity in pig farming, repopulation, surveillance for early detection of ASF, the disposal of ASF infected or suspected pigs, the control of pigs’ and pig products’ movement, the study of ASF epidemiological characteristics and ASF vaccines, cleaning and disinfection, pig slaughtering and consumption management, risk communication, and international collaboration. However, as more than 90% of outbreaks occurred in small- and medium-sized farms with poor biosecurity practices, this was still a great challenge for the prevention and control of the ASF [24]. As a result, within 8–9 months, ASF outbreaks spread rapidly and were reported in all provinces [22].

In the absence of effective ASF vaccines, biosecurity measures have played a critical role in controlling the spread of the disease. The prompt removal of infected pigs and the strict enforcement of movement controls have been essential strategies for preventing the virus from spreading within and between pig farms. However, small-scale pig farms in Vietnam often lack the resources required to implement and sustain effective biosecurity practices, leaving them particularly vulnerable to ASF outbreaks [36,55,56].

**Figure 8:**
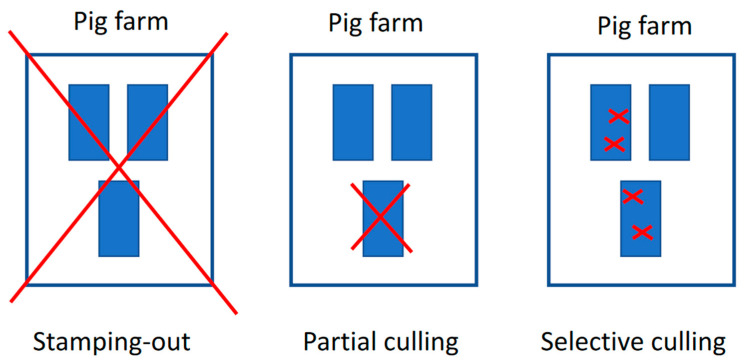
Visual of the total stamping out, partial culling, and selective culling [58].

At the beginning of the ASF outbreak in Vietnam, the rapid detection and complete depopulation of commercial pig farms were employed as an immediate control measure. By the end of 2019, over six million pigs were culled, accounting for more than 25% of the total pig population [57]. However, this approach, combined with the high mortality rate caused by ASF, resulted in the rapid depletion of the national swine population, placing a severe economic burden on pig farmers and disrupting the pork supply chain. In response to these challenges, in July 2019, the MARD amended the ASF control policy to include the possibility of partial and selective culling (also known as spot elimination/removal, “tooth extraction”), which involves the rapid detection and removal of only ASF-infected animals while preserving healthy animals in the herd (official letter No. 5169/BNN-TY dated 22 July 2019). This was carried out with the intent to save resources, reduce the environmental impact of mass carcass disposal, and allow farmers to protect valuable assets, especially valuable breeding pigs [58]. The main differences between these culling approaches are depicted in Figure 8. The success of the spot elimination method in the field has varied depending on several critical factors, including the contagiousness of the ASF virus strain, the availability of strong veterinary infrastructure, accessible and accurate veterinary diagnostics [58], and the implementation of high biosecurity practices on farms. Additionally, the epidemiological situation of the disease within the affected region has significantly influenced the method’s effectiveness. Spot elimination has proven to be a more resource-efficient strategy compared to total depopulation, but its success is contingent on timely detection, robust management, and the farm’s capacity to prevent further spread of the virus [45]. An analysis of the updated control policy revealed that partial culling could, if conducted successfully, save on average over 50% of the total stock with only an eight-day prolongation in implementing control measures. Furthermore, 58% of farms undergoing partial culling scored highly on a time–livelihoods matrix, demonstrating its effectiveness in protecting livelihoods. In contrast, total stamping out did not yield any significant benefits for farmer livelihoods [59].

#### 3.5.2. Ongoing Management of ASF

On 7 July 2020, the Vietnamese government endorsed the “National Plan for Prevention and Control of African Swine Fever, Period 2020 to 2025”. This plan outlined that the overall goal is to proactively monitor for early detection, warning, and the timely and effective application of measures to prevent and control ASF, ensuring the application of biosecurity and disease-free measures in livestock production to minimize economic losses, reducing the adverse impacts of the pork price on the consumer price index (CPI), environment, and trading activities of animals and animal products of Vietnam.

The plan emphasizes a multifaceted approach to tackle ASF. Key solutions include enhancing biosecurity in pig production through isolation, disinfection, and the regulation of feed sources; implementing active and passive disease surveillance to detect and respond promptly to outbreaks; and establishing ASF-free farms and production chains to ensure biosecure environments for domestic and export needs. The plan also focuses on upgrading diagnostic and research facilities, developing ASF vaccines, and strengthening the capacity of veterinary systems. Measures to manage pig movement, slaughter, and disposal aim to prevent disease spread while cleaning and disinfection campaigns target high-risk areas. Public awareness campaigns, international cooperation, and financial mechanisms further support the effective implementation of ASF control strategies. This is the core legislation basis for the localities to develop their own ASF prevention and control strategies based on specific conditions in each province/city to mobilize the necessary resources to deal with ASF outbreaks.

Additionally, while numerous reporting mechanisms exist, electronic real-time reporting platforms offer the fastest solution. In Vietnam, especially following the occurrence of ASF, the DAH has prioritized developing the Vietnam Animal Health Information System (VAHIS). This platform has been used widely in all 63 provinces, completely replacing paper reports or reporting in Excel files via emails. It played a crucial role in enabling policymakers like DAH to propose or introduce mitigation measures on time based on actual situations or by regions or provinces.

#### 3.5.3. Vaccine Research and Deployment in Vietnam

Efforts to research ASF vaccines in Vietnam have been driven by collaborations between the MARD/DAH and the US Department of Agriculture (USDA)/Agricultural Research Service (ARS), and domestic vaccine manufacturers (NAVETCO, AVAC, and DABACO) since 2020. The vaccine candidates leverage attenuated strains of the ASF virus derived from the highly virulent Georgia strain, with genetic modifications designed to improve safety and efficacy [60]. The first vaccine candidate, NAVETCO (NAVET-ASFVAC), is based on the ASFV-G-ΔI177L strain and showed its stability and attenuation following five passages in domestic pigs [61]. This strain is grown on Peripheral Blood Mononuclear Cells (PBMCs) and is recommended for pigs 8 weeks of age. AVAC’s vaccine, AVAC ASF Live, utilizes the ASFV-G-ΔMGF strain, which includes deletions in multigene families MGF360 and MGF505 and is propagated on Diep’s Macrophage Cells (DMAC). This vaccine is indicated for pigs as young as 4 weeks. Lastly, the DABACO developed DACOVAC ASF2, which features the ASFV-G-ΔI177L/ΔLVR strain with deletions in the I177L gene and the Left Variable Region containing nine genes. This candidate, under trial at the time of reporting, uses Plum Island Porcine Epithelial Cells (PIPECs) and is also intended for pigs from 4 weeks of age [62].

The laboratory evaluation of ASF vaccine candidates followed a national standard for ASF vaccine testing to evaluate their sterility, purity, safety, and potency. The National Center of Veterinary Drug and Vaccine Control No. 1 was assigned by DAH to conduct the four-step evaluation of the vaccines [55]. Laboratory testing began with purity and sterility assessments, where real-time PCR and biochemical tests confirmed that all vaccine batches were free from contamination by other pathogens or microbes. Safety testing was conducted by injecting experimental pigs, sourced from disease-free herds, with a 10× dose of the vaccine to observe potential adverse reactions. The NAVET-ASFVAC and AVAC ASF LIVE vaccine trials showed no mortality, clinical signs, or side effects in vaccinated groups. Potency testing measured the antibody production 28 days post-vaccination using ELISA tests targeting the major capsid protein p72. Vaccinated pigs were also challenged with a virulent ASFV (TTKN/ASFV/ĐN/2019 strain, 10^2^ HAD50/pig), and the results showed a 100% survival rate in vaccinated groups [62].

After these studies, the NAVET-ASFVAC and AVAC ASF LIVE vaccines were licensed (marketing authorization) by the DAH in May and July 2022, respectively. However, given the first ASF vaccines, the use of vaccines was directed following two phases: on a small scale, each vaccine was required to be administered 600,000 doses (in at least 50 farms at various scales) under the supervision of Regional Animal Health Offices (RAHOs, belonging to DAH), and the provincial sub-Departments of Animal Health (sub-DAHs) where the vaccines were used; samples were taken to assess the circulation of the vaccine virus and protective immune response (phase 1) [62]. Reports showed that the rate of the protective immune response was 95% and 93.4% for NAVET-ASFVAC and AVAC ASF LIVE vaccines, respectively [62]. Subsequently, on 24 July 2023, the MARD approved these vaccines’ nationwide use and export (phase 2) (official letter No. 4870/BNN-TY). By June 2024, these companies had produced, distributed, and exported nearly six million doses [33].

Additionally, researchers from the Vietnam National University of Agriculture (VNUA) reported the development of two safe and effective live-attenuated vaccine candidates, VNUA-ASFV-LAVL2 and VNUA-ASFV-LAVL3. These vaccines provided complete protection to pigs against virulent contemporary pandemic ASFV infections and demonstrated efficient replication in 3D4/21 cell line. However, a reversion-to-virulence study has yet to be conducted [63,64]. Apart from LAVs, some research groups in Vietnam have studied subunit vaccine candidates. The purified CD2v ED_GCN4pII protein has been shown to elicit both humoral and cellular immune responses in mice, comparable to those induced by the live-attenuated vaccine ASFV-G-∆I177L. This indicates its potential as a promising plant-based subunit ASF vaccine candidate [65].

## 4. Discussion

This review examines the epidemiology, impacts, and management of ASF in Vietnam, which has caused significant disruption to the country’s swine industry since its introduction in 2019. The transition from the epidemic into endemic phases highlights the progress in disease control and ongoing challenges. Key interventions from the National Plan for Prevention and Control of ASF, Period 2020 to 2025, such as enhancing biosecurity measures, applying selective/partial culling, and vaccine development, have reduced outbreaks. However, hurdles like the low adoption rates of ASF vaccines and the emergence of recombinant ASF strains impede ASF control. This study underscores the importance of strengthening the veterinary infrastructure, enhancing farmer compliance, and advancing research to mitigate ASF’s long-term effects on the country’s swine production sector.

When ASF was officially announced in Vietnam in February 2019, the Vietnamese government swiftly implemented comprehensive control measures outlined in the existing national legislation, including an initial total stamping-out policy for affected farms. Simultaneously, the government and provincial authorities mobilized diverse resources while seeking support from international organizations, such as WOAH and FAO, to effectively contain and prevent the spread of the disease. Despite these efforts, ASF prevention and control remain a significant challenge due to the complex nature of the ASFV, limited knowledge about its transmission routes and risk factors, and the high prevalence of outbreaks (over 90%) occurring in small- and medium-sized farms [24]. These farms often lack the resources, infrastructure, and awareness needed to implement effective biosecurity measures, leaving them highly vulnerable to ASF transmission and undermining the success of national control strategies. As a result, by the end of 2019, more than 8500 outbreaks had been reported, with over six million pigs dead or culled because of the disease.

Managing multiple ASF outbreaks simultaneously placed an overwhelming burden on veterinary authorities. This burden is due to the substantial resources required for implementing the total culling policy [58]; veterinary services at the commune and village levels are especially often underfunded, poorly staffed, and inconsistently trained. Local animal health workers, who play a vital role in disease detection and response, are lacking in many areas due to administrative restructuring and funding shortfalls. This absence of public veterinary services leads to delays in diagnosing and reporting ASF cases, which, in turn, contributes to the spread of the disease. Moreover, the limited diagnostic capacity of veterinary laboratories hinders the accurate identification of ASF, further complicating outbreak management [26].

Effective disease control also requires the systematic monitoring and real-time recording of livestock movement patterns, which is currently lacking. One of the significant challenges identified in ASF management in Vietnam is the lack of reliable data on livestock movement. Limited animal movement data have been available in some provinces, but the accuracy and quality of this information are not well-known [26]. This gap is particularly problematic for smallholder farms, which represent the vast majority of pig farming operations in Vietnam and are characterized by low levels of biosecurity. These farms are highly vulnerable to ASF outbreaks, and their limited resources make implementing robust prevention measures difficult. However, the Law on Animal Health mandates that animals and animal products undergo quarantine at the point of origin before being transported out of the provincial-level area. Consequently, utilizing an online tracking system could be an effective way to manage data on animal movement. This information is vital for tracing the source if a disease is detected in transported animals.

Farmers’ behaviors further complicate ASF management efforts. Many smallholder farmers delay reporting suspected ASF cases due to several interlinked issues. First, farmers fear losing their entire herd if even one infected pig is noted, as infected herds are often culled to prevent further spread. Second, compensation rates are low and inconsistent, and the process of receiving financial reimbursement is plagued by lengthy delays and bureaucratic hurdles [54]. Farmers sometimes wait months or even years to receive compensation, creating a disincentive to report infections. Third, the symptoms of ASF can mimic other diseases, such as classical swine fever [36], making diagnosis challenging at the farm level. Finally, the potential reputational damage within local communities deters farmers from acknowledging outbreaks on their farms, leading to further delays in disease control efforts [22]. Reports have also indicated that some small-scale pig-raising households disposed of pig carcasses in unsecured locations, such as rivers and canals near densely populated areas, significantly increasing the risk of further spreading the disease [17]. Additionally, practices such as swill feeding remain common, further increasing transmission risks and leading to a considerable challenge to control and prevent the spread of ASF. Conversely, large-scale commercial farms with stringent biosecurity protocols have largely been spared from ASF outbreaks [22]. These farms often employ advanced measures such as controlled access, regular disinfection, and strict animal health monitoring, significantly reducing the risk of virus introduction and transmission. This stark difference highlights biosecurity’s critical role in disease prevention and management.

The live attenuated ASF vaccines are expected to provide more tools for controlling ASF in Vietnam. The vaccine trials conducted across the country have yielded promising results. However, appropriate monitoring and surveillance are needed when applying ASF live-attenuated virus-based vaccines to control ASF in the field [66]. Because of their nature, LAVs might induce, under certain circumstances, mild or severe negative reactions. For that reason, evaluating the probability of those negative reactions occurring, developing protocols for the national veterinary services to identify if negative reactions are occurring, and communicating this information clearly to producers and practitioners are important in order to set appropriate expectations when implementing a control plan. Additionally, administering the vaccine to pigs infected by or exposed to the ASFV may increase the chances of the negative effects and the animalization of the vaccine, and, thus, official veterinary services should set up mechanisms to certify and verify the use of the vaccine according to standard protocols. Moreover, vaccine platforms are being evaluated only for weaned piglets. Depending on the platform, the recommendation is to inoculate >4-week or >8-week-old piglets, and the vaccination of sows, gilts, and boars is not recommended. Therefore, any control plan considering using ASF vaccines should also establish mechanisms to monitor the use of vaccines, ensuring following the protocol and instructions of the manufacturers. Moreover, strategies to protect sow farms, boar studs, and genetic nuclei should be considered. At least for now, the protection of those types of farms seems to be related to the best practices of biosecurity and disinfection. Depending on the structure of the farms, a separation between vaccinated and unvaccinated populations (which certainly should occur given the attributes of the LAVs described here) may be challenging to implement. Proper monitoring and surveillance are essential when deploying ASFV live-attenuated virus-based vaccines for ASF control in endemic countries [66].

A strong regulatory framework and definitions of strategies are required. Certainly, ASF vaccines currently being evaluated might not be considered a “silver bullet”. Assuming that field studies are successful in demonstrating safety and efficacy, countries will be required to implement a strong regulatory framework to supervise how and when the vaccines are being used, and that protocols are being followed correctly, design plans for vaccinated and unvaccinated populations, and monitor the circulation of wild strains, and the success of vaccination campaigns. It is very likely that, according to international standards, vaccinated populations (and unvaccinated populations in which contact with vaccinated animals cannot be ruled out) may not be able to access markets that are free from the disease, at least through the duration of the precautionary measures, unless international regulations are revised, which seems to be unlikely in the short term. In this scenario, the most important goal of countries intending to use the ASF vaccine should be to mitigate the impact of disease spread, particularly in smallholders, protect genetic resources, and prevent and protect food security in affected countries.

In addition, several challenges persist that need to be taken into consideration. These include farmers’ hesitance to adopt new vaccines [67], the competition from inferior products, a limited application to growing pigs rather than breeding stock, and the emergence of recombinant ASF strains, particularly those combining genotypes I and II [68,69]. The lack of publicly available data on the long-term safety and efficacy of the vaccines has led to concerns about the possibility of a reversion to virulence [67]. Other challenges, such as vaccine virus shedding, vertical and horizontal transmission, immunogenicity against various field strains, a potential recombination with field viruses, and post-vaccine complications, should be considered in vaccine development. These concerns are especially critical for LAVs [67]. Therefore, compliance with the vaccine protocol and manufacturer’s instructions is essential. The lack of a Differentiating Infected from Vaccinated Animals (DIVA) feature in current vaccines complicates surveillance efforts, as it is difficult to distinguish between infected and vaccinated pigs [70,71,72]. Additionally, smallholder farms often lack resources and biosecurity measures, limiting the impact of vaccination programs [55,73].

Models have estimated that vaccination coverage ranging from 35.71% to 49.70% (small herds may need up to 80% coverage) may be needed to provide herd immunity, depending on the farm scale [43]. However, field data are limited, and more work is needed to help the government design strategies to ensure effective control. The concept of herd immunity remains uncertain for ASF due to its unique transmission dynamics, which differ from other viral diseases. Since ASF can persist in the environment and spread through direct contact, fomites, and wild reservoirs, specific percentage estimates for herd immunity may be unreliable unless backed by comprehensive epidemiological modeling.

As ASF is known to cause significant social and economic impacts, it is essential that we gain a deeper understanding of the socioeconomics of the disease and the pig and pork value chains, particularly in low-biosecurity settings [74]. Understanding farmer perceptions, attitudes, and decision-making processes is key to designing effective education and outreach programs. This includes examining the economic losses incurred by smallholder farmers, the disruptions in pork supply chains, and the broader implications for food security and livelihoods. However, few studies have been conducted in this field in Vietnam, with limited scopes in some areas. Identifying risks posed by pig value chains and proposing appropriate mitigation measures [75] would be useful, especially for veterinary authorities. Additionally, targeted research into stakeholders’ financial challenges and behavior within low-biosecurity systems could aid in designing more effective and equitable control strategies. Addressing behavioral barriers, such as a reluctance to report outbreaks or adopt biosecurity measures, will improve compliance and cooperation in disease management.

As ASF transitions from an epidemic to an endemic phase, research should focus on sustainable strategies to coexist with the disease while minimizing economic losses. Developing frameworks for continuously monitoring and evaluating ASF control measures will ensure adaptability and effectiveness over time. These efforts will contribute to building a resilient swine industry capable of managing future ASF challenges.

## 5. Conclusions

In the six years since its introduction, the ASF epidemic has profoundly altered Vietnam’s swine industry, accelerating the decline of small-scale household farms and promoting the growth of professional and large-scale operations. Despite progress in controlling the disease, challenges such as the high proportion of households and small-scale farms with low levels of biosecurity, a shortage in veterinary personnel at localities, difficulties in the implementation of compensation policy, resource limitations, the low adoption rates of ASF vaccines, and the persistence of ASFV recombinant strains continue to hinder eradication. Strengthening reporting and surveillance systems, enhancing the application of biosecurity measures, especially for smallholder farms, and advancing vaccine research are essential for the sustainable management of ASF in the country.

## Figures and Tables

**Table 1:** Summary of the ASF outbreaks from 2019 to 2024.

Year	Number of Outbreaks	Number of ASF-Affected Provinces/Cities	Number of Pigs Dead/Culled (Heads)
2019	8517	63/63	6,000,000
2020	1808	50/63	87,668
2021	3211	60/63	299,878
2022	1407	54/63	66,715
2023	952	46/63	44,390
2024	1669	48/63	92,707
Total	17,564		6,591,358

**Table 2:** Summary of studies related to the transmission of ASFV in Vietnam.

Study Locations	Methods	Daily Transmission Rate (*β*)	Basic Reproduction Number (R_0_)	Reference
An experimental study in Vietnam	Susceptible–Infectious–Removed (SIR) models, EG, ML	0.729 (95% CI: 0.379–1.765) for EG; 1.004 (95% CI: 0.283–2.450) for ML	2.916 (EG), 4.015 (ML)	Oh et al., 2023 [46]
In five provinces in Northern Vietnam	SIR models	0.094 (95% CI: 0.092–0.096) to 0.30 (95% CI: 0.29–0.31).	1.41 to 10.8 for the infectious period (15, 19 and 30 days).	Mai et al., 2022 [44]
Two commercial farms (Northern Vietnam)	SIR models, ML, bootstrap	NA	3.8–4.76 for fattening pigs, significantly higher than sows (1.55–1.78)	Mai et al., 2022 [43]
Ten private farms on different scales (Northern Vietnam)	SIR models, EG, ML, AR	0.16 (95% CI: 0.12–0.22) to 0.37 (95% CI: 0.20–0.63)	1.49 (95% CI: 1.05–2.21), 1.58 (95% CI: 0.92–2.56), and 1.46 (95% CI: 1.38–01.57) for the EG, ML, and AR, respectively	Mai et al., 2022 [45]

## Data Availability

The data are available upon request.
